# Strain Rate Effect upon Mechanical Behaviour of Hydrogen-Charged Cycled NiTi Shape Memory Alloy

**DOI:** 10.3390/ma14164772

**Published:** 2021-08-23

**Authors:** Fehmi Gamaoun

**Affiliations:** 1 Department of Mechanical Engineering, College of Engineering, King Khalid University, Abha 61421, Saudi Arabia; fgamaoun@kku.edu.sa; 2 Laboratory of Mechanics of Sousse, National Engineering School of Sousse, University of Sousse, Sousse 4054, Tunisia

**Keywords:** shape memory alloys, superelastic, hydrogen, cyclic loading, martensite variants

## Abstract

The rate dependence of thermo-mechanical responses of superelastic NiTi with different imposed strain rates after cycling from 1 to 50 cycles under applied 10^−5^s^−1^, 10^−4^s^−1^ and 10^−3^s^−1^ strain rates, immersion for 3 h and ageing has been investigated. The loaded and unloaded as-received NiTi alloy under an imposed strain of 7.1% have shown an increase in the residual deformation at zero stress with an increase in strain rates. It has been found that after 13 cycles and hydrogen charging, the amount of absorbed hydrogen (291 mass ppm) was sufficient to cause the embrittlement of the tensile loaded NiTi alloy with 10^−5^s^−1^. However, no premature fracture has been detected for the imposed strain rates of 10^−4^s^−1^ and 10^−3^s^−1^. Nevertheless, after 18 cycles and immersion for 3 h, the fracture has occurred in the plateau of the austenite martensite transformation during loading with 10^−4^s^−1^. Despite the higher quantity of absorbed hydrogen, the loaded specimen with a higher imposed strain rate of 10^−3^s^−1^ has kept its superelasticity behaviour, even after 20 cycles. We attribute such a behaviour to the interaction between the travelling distance during the growth of the martensitic domains while introducing the martensite phase and the amount of diffused hydrogen.

## 1. Introduction

NiTi Shape Memory Alloys (SMAs) have been employed on a large scale within various engineering applications such as Micro-Electro-Mechanical Systems (MEMS), aerospace structures [1,2], and biomedical applications [3,4]. The success of these applications hinges on their capability of having an excellent combination of several properties, including superelasticity, corrosion resistance, and biocompatibility. Moreover, due to its larger flexibility, the equiatomic superelastic NiTi archwire turns to be one integral part of orthodontic treatment because of exerting a relatively constant force in the range of tooth movement. However, the NiTi alloy is found to be very sensitive to the hydrogen diffusion caused by adding fluoride to prophylactic agents and toothpaste in the oral cavity [5,6,7]. In fact, the diffusion of hydrogen is a main environmental cause for premature fractures of the superelastic NiTi alloys [8,9].

We have shown, after immersion, that the NiTi alloy fractures in a brittle manner for different strain rates [10,11] and for varied immersion time [12,13]. In fact, at room temperature and after immersion in a 0.9% NaCl aqueous solution, while utilizing a varied current density of 5, 10, and 20 A/m^2^ from 2 to 24 h, embrittlement takes place after ageing at room temperature rather than immediately after hydrogen charging. This behaviour suggests that when hydrogen diffuses to an entire alloy, not concentrated just close to the surface, it can most likely obstruct the stress-induced martensite phase (B19 phase). Ogawa et al. [14] showed by Thermal Desorption Analysis (TDA); i.e., in case the diffused hydrogen exceeded 50–200 mass ppm, that the mechanical SMA characteristics could change, hence the appearance of premature failure. The superelastic NiTi archwire embrittlement, after hydrogen-absorption, was greatly linked to the transformation of stress-induced austenite-martensite [15]. This behaviour was attributed to the accumulation of hydrogen atoms, preferentially within regions that contain continual deficiencies, because of being trapped by retained martensite plates and dislocations, forming an obstacle which would cause fracture when the martensite domains were forced to pass across these boundaries [11]. In several studies [16,17], permanent defects could be obtained by cyclic deformation. In [18], the authors indicated that the pseudo-elastic hysteresis loops would decrease as the number of cycles increased and the strain hardening slope grew. B. Kockar et al. [19] mentioned that the main reason for the cyclic instability of the NiTi SMAs’ pseudoelastic response was the local plastic accommodation of incompatibility between the B2 parent phase and the B19 phase during transformation. The authors in [20] indicated that during thermomechanical cycling, the variant interfaces would move backward and forward, while transformation-induced defects such as dislocations would build up and obstruct any interface mobility in the subsequent cycles. As a result, accumulations of retained martensite domains and local residual stresses would eventually affect the macroscopic superelastic behaviour of the NiTi alloy. In addition, we note that the effect of the strain rate on the cyclic deformations of superelastic NiTi alloys was studied by some experimental connotations [21,22,23]. It was found that rate dependence mainly resulted from the interaction between internal heat production (due to the mechanic dissipation and transformation latent heat) as well as the heat exchange with the external environment. Moreover, during cycling, the higher the strain rate the bigger the number of martensite domains in the austenite-martensite transformation.

Therefore, as the NiTi archwires are subjected to varied mechanical cycling before insertion in the oral cavity, this study aims to determine the hydrogen effect on tensile tests of the superelastic NiTi alloy under different imposed strain rates. The effect of the residual deformation after cycling with 10^−5^s^−1^, 10^−4^s^−1^ and 10^−3^s^−1^ before immersion and tensile testing will be discussed.

## 2. Experimental Procedure

A commercial superelastic Ni-Ti (50.9 (at.%) Ni–49.1 (at.%) Ti, from Forestdent Co.), is used in this study. We obtained the heat treatment material’s transformation temperature from the differential scanning calorimetry test at a scan rate of 10 °C/min. The start and finish temperatures of the martensite transformation (M_s_ and M_f_) are equal to −8 and−26 °C, respectively; and the start and finish temperatures of the austenite transformation (A_s_ and A_f_) are equal to −11 °C and −4 °C, respectively. The initial Young’s modulus of the austenite and martensite phases were, respectively, 24 GPa and 12 GPa. During the mechanical tensile tests, we used rectangular specimens that had a gage length of 20 mm and across-sectional dimension of 0.64 × 0.43 mm^2^.

**Figure 1:**
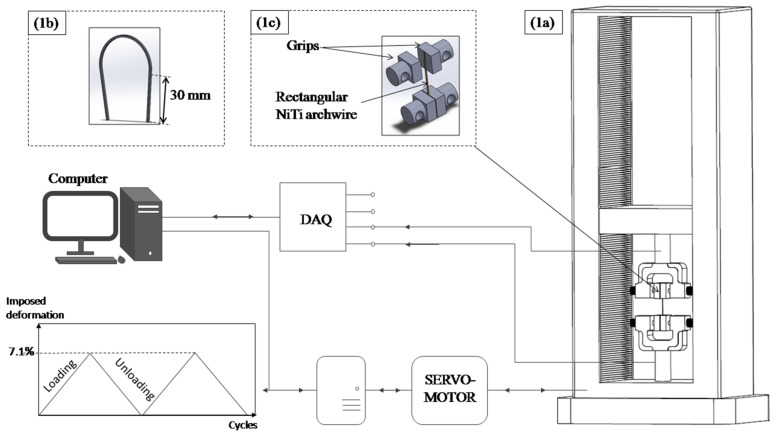
Loading protocol of the monotonic and cyclic loading: (**a**) Schematic representation of the Instron tensile machine, (**b**) used part of the as-received alloy, and (**c**) clamping method of the NiTi archwire.

Cyclic tensile tests, from 1 to 50, and a uniaxial tensile test at room temperature were carried out on a standard Instron screw-driven mechanical testing 5566-type machine (Figure 1a). With the aim of studying the loading rate influence up on the NiTi archwire’s superelastic behaviour, we utilized the10^−5^s^−1^, 10^−4^s^−1^ and 10^−3^s^−1^ strain rates. The imposed strain of 7.1% was performed for the applied cyclic tensile test. In order to run the test, 30 mm was cut from the commercial superelastic NiTi archwire (Figure 1b) and clamped between the bottom and top flat grips of the Instron machine (5 mm distance) (Figure 1c). The strain was measured as across-head overall displacement in addition to a 20 mm initial gauge length. A brand-new specimen was used after each monotonic or cyclic test.

**Figure 2:**
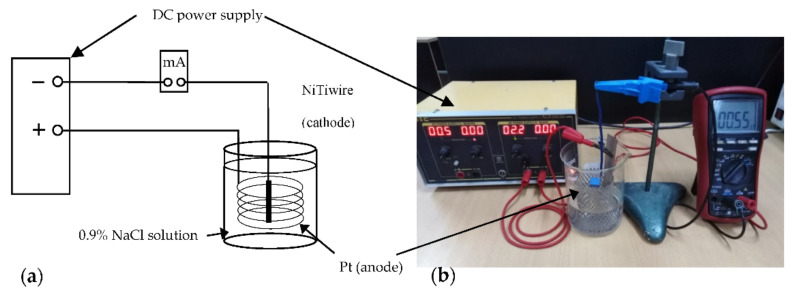
(**a**) Scheme of hydrogen charging (**b**) setup by electrolysis.

Specimens’ hydrogen charging was done through the immersion in a 0.9% NaCl solution. This was also performed for 3 h at room temperature at a 10 A/m^2^ current density. A Direct-Current (DC) power supply was used for immersion, as shown in Figure 2. Circular platinum and the NiTi archwire were based on the 0.9% NaCl solution as the anode and the cathode, respectively. The current density controlled the generated gaseous hydrogen on the surface of the sample. Before immersion, samples were polished with a No. 600-grit SiC paper. After that, it was ultrasonically washed for 5 min in acetone. To homogeneously distribute the diffused hydrogen after immersion, we would age the specimens for one day in air at room temperature [24,25].

After immersion for 3 h and ultrasonic cleaning with acetone for 5 min, the amount of desorbed hydrogen was measured by TDA through the use of a gas chromatograph. This latter was actually calibrated under 10^−6^ Pa of vacuum with a standard mixture of argon gases and hydrogen. Moreover, we employ the high-purity argon as a carrier gas. TDA was performed on a 20 mm NiTi alloy total length. The sampling time of the carrier gas to the gas chromatograph was 5-min intervals at a heating rate of 100 °C/h. All TDA examinations were carried out after 24 h hydrogen charging. The total amount of hydrogen within the specimen was determined to be the sum of desorbed hydrogen within peaks of a hydrogen desorption rate [26,27]. At least two analyses have been done for each cyclic condition, and the experimental errors of the absorbed-hydrogen amount have been estimated between 5% and 10%.

## 3. Results and Discussion

**Figure 3:**
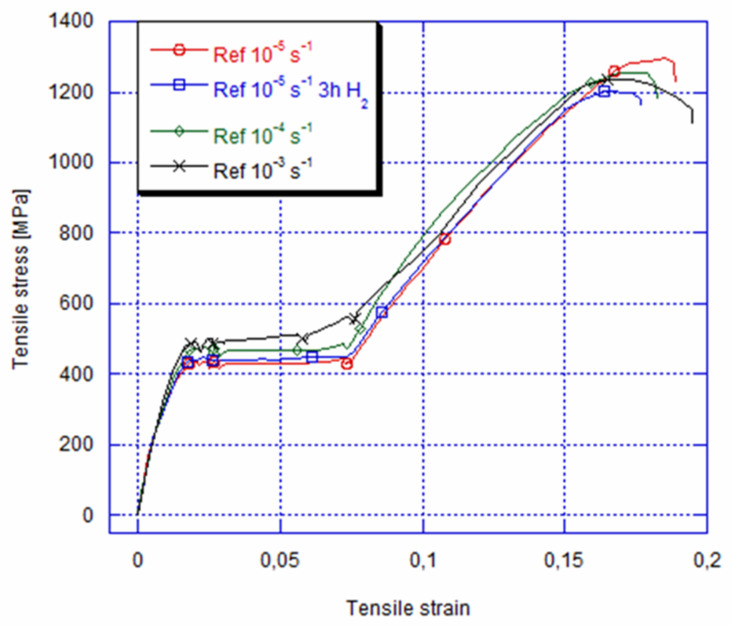
Typical engineering stress–strain curves of superelastic Ni-Ti alloy immersed for 3 h and aged for 24 h at different strain rates of 10^−5^s^−1^, 10^−4^s^−1^ and 10^−5^s^−1^ and as-received at 10^−5^s^−1^.

The non-charged and charged specimens’ tensile stress–stain curves for 3 h with the current density of 10 A/m^2^ besides annealing for 24 hat room temperature are shown in Figure 3. For strain rates of a non-charged hydrogen specimen between 10^−5^ and 10^−3^s^−1^, the elastic loading of an austenite parent phase is observed, which is followed by “a plateau’’ during the austenite-martensite transformation. After that, there follows the elastic deformation of a martensite phase until fracture. It can be seen that the martensite start stress goes up from about 430 MPa to 460 as well as 490 MPa for a strain rate of 10^−5^s^−1^ to 10^−4^s^−1^ and 10^−3^s^−1^, respectively. On the other hand, for the 10^−3^s^−1^ strain rate, the strain hardening effect of the austenite-martensite transformation goes up.

**Figure 4:**
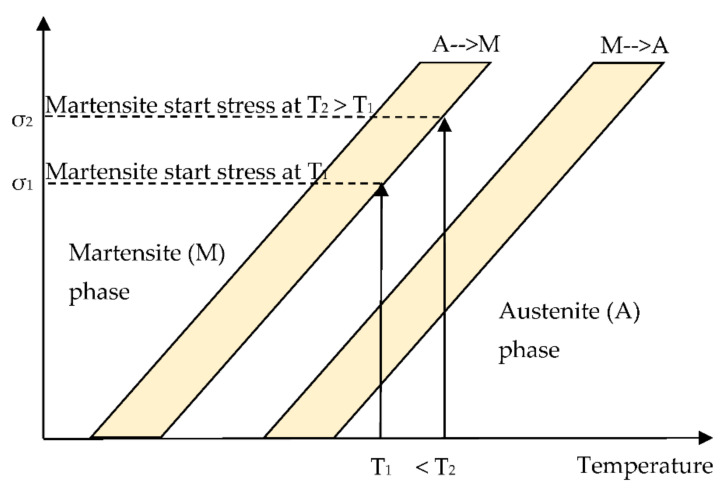
Simplified NiTi phase diagram showing the increase in the martensite starting stress from σ_1_ to σ_2_ when the temperature goes up from T_1_ to T_2__,_ respectively.

The strain rate dependency appears in a clear way during the austenite martensite transformation with the growth of the ‘‘plateau’’ slope. After that, it increases in the martensite start stress. Such a rise could be interpreted by the material’s viscoelastic behaviour, and this is because of the friction between the martensite interface variants as well as the strain rate dependency that is actually caused by a thermomechanical NiTibehaviour due to the fact that the austenite-martensite transformation was exothermic [28,29]. Indeed, when the strain rate goes up, an internal heat production is as fast as the strain rate and the latent heat of the specimen is confined in the alloy. Consequently, a quantity of heat cannot be transferred outside of the NiTi alloy since the conduct time is shorter. This enhancement in temperature leads to a rise in the necessary applied load for inducing the martensite phase (Figure 4). Thus, both the amount of the required stress inducing the martensite and the slope of the austenite-martensite transformation will increase [17,30,31].

After 4 h of immersion with a 10 A/m^2^ current density, Figure 3 illustrates a typical tensile curve for the applied 10^−5^s^−1^ strain rate. This result shows that the material has conserved its superelastic behaviour and that there has not been any fracture detected. However, compared to the non-immersed alloy in the 0.9% NaCl solution, the NiTi archwire indicates arise in the martensite start stress of about 10 MPa. This phenomenon is attributed to the solid-solution hardening during hydrogen charging [18,19,20,21,22,23,24,25,26,27,28,29,30,31,32].

**Figure 5:**
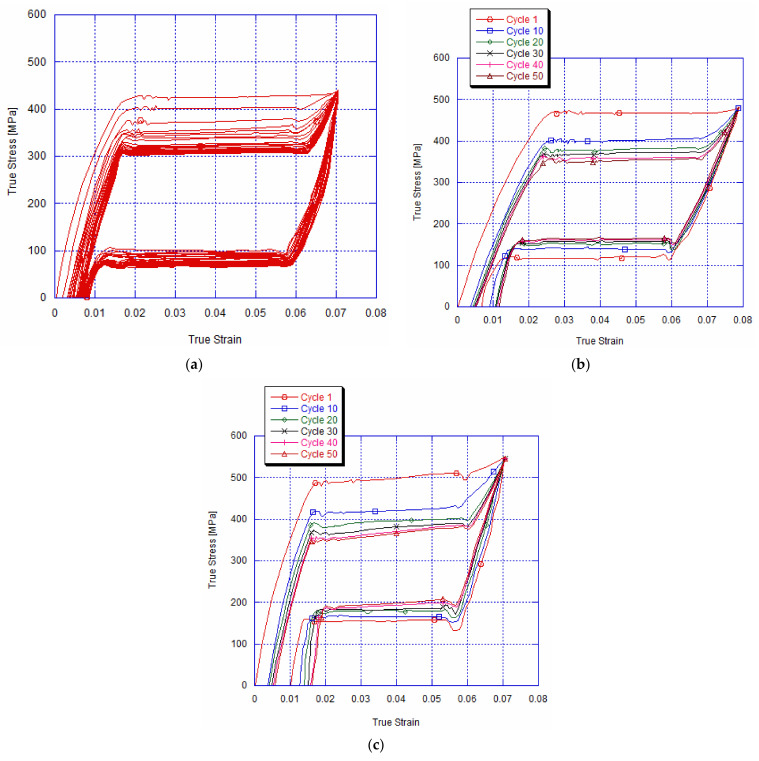
Typical strain cycling curve after 50 cycles for deformed specimen until 7.1%, at imposed strain rate of (**a**) 10^−^^5^s^−1^, (**b**) 10^−^^4^s^−1^ and (**c**) 10^−^^3^s^−1^, showing reduction in phase-transformation yield stress and increase in residual strain.

Figure 5a depicts the tested specimen’s stress–strain curves for 50 cycles at the lowest 10^−5^s^−1^ strain rate under an imposed strain of 7.1%. This latter represents the end of the plateau of the austenite-martensite transformation of the as-received NiTi archwire (Figure 3). The result highlights that the slope of the austenite-martensite transformation increases in a progressive manner during cycling. On the other hand, the martensite start transformation decreases with the growth of the loading and unloading cycles. This martensite start stress decreases from approximately 430 MPa at cycle 1 to about 310 MPa at cycle20. We can attribute this reduction to the introduction of dislocations and to the residual martensite phase during cycling, which is the main cause for establishing the residual stress [20,33]. Therefore, this residual stress assists the stress-induced transformation during the next loading by enabling martensite bands to activate at a lower macroscopic stress. Cyclic loading and unloading with imposed 10^−4^s^−1^ and 10^−3^s^−1^ strain rates are represented by Figure 5b,c, respectively. It is clear that the slope of the austenite martensite transformation plateau increases steadily with the number of cycles, besides raising the imposed strain rate. In addition, after 10^−4^s^−1^ and 10^−3^s^−1^ of imposed strain rates, the aforementioned yield stress falls approximately by 80 MPa and 110 MPa, respectively, after 20 cycles of loading and unloading. These results show that the internal residual stress becomes more significant with the increase in the imposed strain rate.

**Figure 6:**
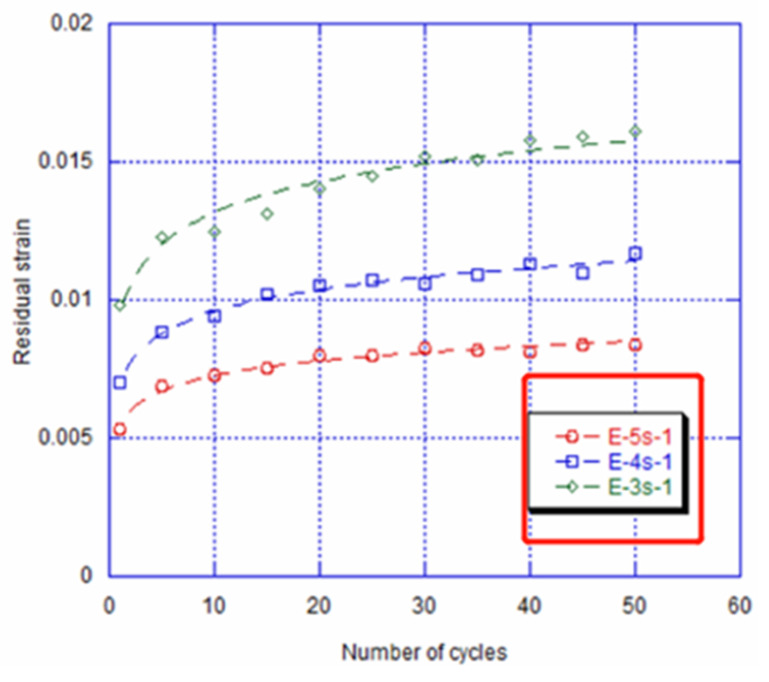
Evolution of residual strain at zero stress as a function of number of cycles for cyclic deformed specimen with 7.1% strain after different imposed strain rates of 10^−^^5^s^−1^, 10^−^^4^s^−1^ and 10^−^^3^s^−1^ (represents the fitting curve).

Figure 6 depicts the residual strain evolution as a function of the number of cycles for the three various strain rates. This residual strain is significant in the first cycles and goes up progressively according to the number of cycles. Thus, after 20 cycles, for the imposed strain rate of 10^−3^s^−1^, the residual deformation at zero stress is almost double of the value obtained with 10^−5^s^−1^. According to these results, we can deduce that there is an interaction between the decrease in the martensite start stress and the increment or increase in the residual strain at zero stress, and that there is an imposed strain rate during cycling. The interaction between the critical stress for introducing the martensite and the increase in the residual strain at zero stress was studied previously [20]. In fact, during thermo-mechanical cycling, the phase boundaries propagate forward and backward [34]. Throughout this reversible transformation, defects such as dislocations will form and act as an obstacle for the interface movement for the next cycles. Nevertheless, Chu K et al. [35] showed that during the fatigue tests of NiTi micropillars, the nanosized residual martensite domains, which were in fact blocked by the dislocations, could decrease in a considerable way the overall transformation stress throughout a generated remanent stress and the direct variant increase, and that was without overcoming the martensite nucleation obstacle. Similarly, Kan Q. et al. [36] studied the effect of the strain rates upon loading or unloading the SMA NiTi alloy. They found that the residual strain was accumulated by raising the number of cycles and strain rates. Indeed, after 20 cycles, the residual strain was about 2.04% for a 3.3 10^−^^4^s^−1^ strain rate and it reached 4.2% with 3.3 10^−^^2^s^−1^. In addition, they found that the NiTi SMAs’ super-elasticity degradation after cycling was caused by interacting between the dislocation slipping and the martensitic transformation. Moreover, they concluded that the super-elastic NiTi SMAs’ dislocation slipping could be generated through a high local stress close to the austenite-martensite interfaces. This was in general during the repeated martensitic transformation as well as during its reverse [37,38,39]. Consequently, during the cyclic deformation, the dislocation density and then the internal stress of NiTi SMAs went up rapidly.

In our case, a decrease in the martensite start stress and an increase in the residual strain at zero stress can be explained by the rise in the dislocation density and then the internal stress, which acts as an obstacle for the growth of the martensite bands and the nucleation of new variants. In addition, during the reverse transformation, some of the martensite bands can be pinned by dislocations and remain untransformed after unloading [36,40]. During the NiTi specimen’s cyclic deformation, the temperature variation is based upon interacting between the heat conduct and the internal heat production. This can greatly depend on the strain rate. In the condition that the strain rate is low, the convected and conducted heat transfer is quicker than the heat production, and this is because of the latent heat and inelastic dissipation. When a strain rate is high, the inelastic-dissipation and latentheat production is quicker than the heat transfer. As the critical stress of the introducing martensite increases linearly with the rising test temperature [24], the required driving force of dislocation slipping will become higher, and then the density of dislocation will build up [34]. As a result, the increase in the residual stress with the strain rates is due to the higher density of dislocation. Additionally, the bigger the number of cycles, the more accentuated the internal stress and the retained martensite by dislocation will be. This is done by raising the dislocation density after the reverse transformation.

**Figure 7:**
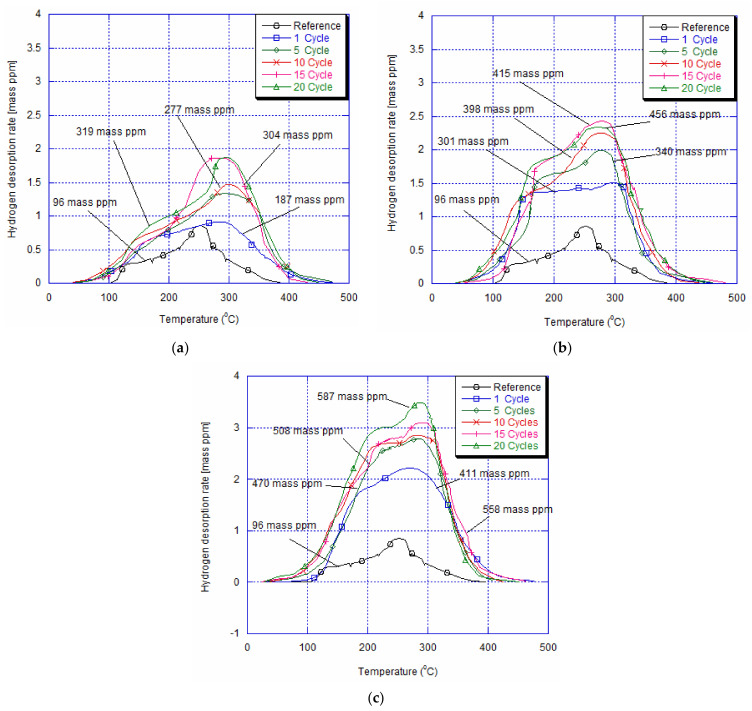
Hydrogen thermal desorption curves for specimens immersed for 3 h and aged for 24 h at room temperature after 13 cycles with imposed deformation of (**a**) 10^−5^s^−1^, (**b**) 10^−4^s^−1^ and (**c**) 10^−3^s^−1^, showing an increase in the amount of absorbed hydrogen with stain rates.

The amounts of absorbed hydrogen by the NiTi superelastic alloy obtained by TDA after 3 h of immersion of hydrogen charging within the 0.9% NaCl solution under an imposed current density of 10 A/m^2^ at room temperature are illustrated in Figure 7. This quantity of diffused hydrogen is calculated by subtracting the amount of pre-dissolved hydrogen in the as-received NiTi archwire (8 mass ppm). Before mechanical testing and after 3 h of immersion, the as-received specimen shows approximately 96 mass ppm. The maximum peak of desorption is obtained at about 244 °C. Moreover, a shoulder of the desorption peak appears at about 142 °C. The TDA after loading and unloading from 1 to 20 cycles with 10^−5^s^−1^ and 10^−4^s^−1^ of the imposed strain rate and hydrogen charging is represented in Figure 7a,b, respectively. We can see that after one cycle and immersion, the amount of absorbed hydrogen is about 187 mass ppm with the lower strain rate, and it grows up to 301 mass ppm after cycling with an imposed deformation of 10^−4^s^−1^. Besides, the diffused hydrogen into the NiTi archwire increases progressively with the number of cycles of loading and unloading at the imposed strain of 7.1%. After 20 cycles and 3 h of electrolytic charging in 0.9% NaCl solution for the lower strain rate, this value is about 319 mass ppm, and it becomes 456 mass ppm at 10^−4^s^−1^. We can notice that for both strain rates, the primary peak temperature of hydrogen is in the rage of 270–300 °C, which is quite higher than the as-received and charged by hydrogen specimen. According to Yokoyama et al. [41], this behaviour is due to the fact that the hydrogen states can vary from a parent phase to a martensite one. Additionally, it is remarkable that the shoulder of the desorption peaks, between 145 and 175 °C, is higher than the as-received and charged specimen. Concerning the imposed 10^−3^s^−1^ strain rate, the quantity of absorbed hydrogen after one cycle and immersion can be about 411 mass ppm (Figure 7c). This value is almost double of the one obtained with an imposed strain rate of 10^−5^s^−1^ and four times higher than the as-received and hydrogen-charged alloy. In addition, after 20 cycles, we notice that the quantity of hydrogen is about 587 mass ppm, which is almost double of the measured value with the lower strain rate obtained after the same number of cycles. Similar to the results obtained with 10^−5^s^−1^ and 10^−4^s^−1^, the maximum absorbed peak remains in the same range of temperature, from 270 to 300 °C, hence the similarity between hydrogen states and/or trapping. However, a shoulder of the desorption peak is affected by the imposed strain rate. Indeed, this peak is increased and appears in the range 200–230 °C. T. Shimada et al. [42] indicated that the shoulder of the hydrogen desorption peak of a superelastic NiTi alloy was lower than that for the specimen charged with an applied stress of 600 MPa. The authors concluded that this phenomenon was due to the formation of the martensite phase under the applied stress where the diffusion distance and hydrogen trapping sites were different from the parent phase. On the other hand, an increase in the shoulder would be in fact detected for the NiTi alloy, which was charged by hydrogen in the martensite phase presence and under a static applied strain [43]. The authors mentioned that the diffused hydrogen tended to form unstable states. After a dynamic cyclic tensile test, carried out under the stress plateau region, the unstable hydrogen states would accumulate, leading to a hydrogen desorption peak shoulder [8]. The latter would increase by raising the strain rate as well as the number of deformation cycles.

**Figure 8:**
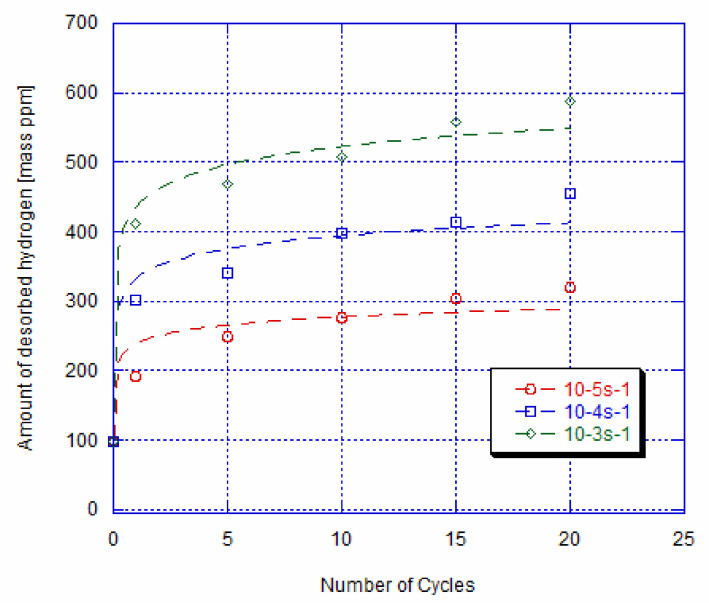
Amount of absorbed hydrogen vs. number of cycles of loaded and unloaded specimens at imposed 10^−5^s^−1^, 10^−4^s^−1^, and 10^−3^s^−1^ strain rates and hydrogen charging for 3 h (represents the fitting curve).

The evolution of the mean value of the amount of absorbed hydrogen as a function of cycles number at different imposed strain rates, i.e., 10^−3^s^−1^, 10^−4^s^−1^, and 10^−5^s^−1^, is depicted in Figure 8, respectively. It is seen that there is a direct relation between the amount of absorbed hydrogen, the imposed strain rate, and the number of cycles. Moreover, this behaviour is in coherence with the residual strain evolution at zero stress as a function of the number of cycles observed in Figure 6. In fact, as explained previously, the increase in the residual deformation with the strain rate and the number of cycles is caused by the rise in the internal stress due to the density of dislocations and to the pinned martensite by dislocations after unloading. The dislocations and the retained martensite represent a preferential site for hydrogen diffusion rather than the parent phase. In fact, the diffusion of hydrogen contributes to the stress relaxation of the infinite dislocation strain at the core. Additionally, the amount and diffusion rate of hydrogen are higher in the martensite phase in comparison with those in the austenite phase [41]. Consequently, we can deduce that the amount of absorbed hydrogen goes up with the increase in the residual strain at zero stress, since the density of the dislocation and the quantity of the retained martensite grow after each cycle.

**Figure 9:**
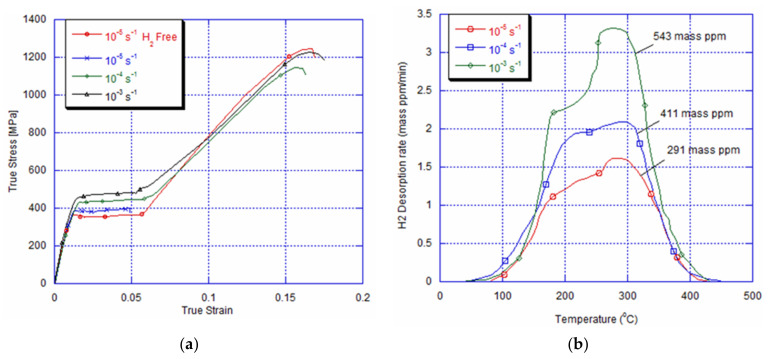
(**a**) Tensile curve at 10^−5^s^−1^ showing fracture after 13 cycles with imposed strain rate of 10^−5^s^−1^, and (**b**) amount of absorbed hydrogen after the same number of cycles at 10^−5^s^−1^, 10^−4^s^−1^, and 10^−3^s^−1^.

**Figure 10:**
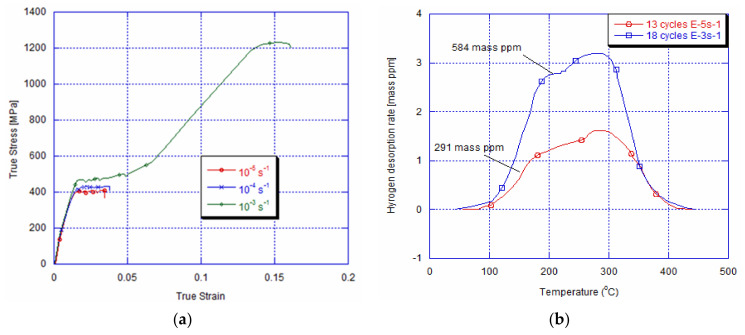
(**a**) Tensile curves obtained after 18 cycles and immersion for 3 h showing the embrittlement of loaded specimen with imposed strain rates of 10^−5^s^−1^ and 10^−4^s^−1^ and superelastic behaviour of loaded specimens with 10^−3^s^−1^, and (**b**) comparison between critical amount of absorbed hydrogen causing fracture at 10^−5^s^−1^ and quantity of absorbed hydrogen after 18 cycles with 10^−3^s^−1^ and immersion.

Figure 9a shows the NiTi archwire’s stress strain behaviour for the three quasi-static strain rates after 13 cycles with a strain rate of 10^−5^s^−1^ and immersion for 3 h at room temperature. It can be seen that for a lower strain rate of 10^−5^s^−1^, the NiTi alloy is embrittled by hydrogen charging. Indeed, the failure stress is detected in the plateau of the martensite start stress; whereas, the tested 10^−4^s^−1^ and 10^−3^s^−1^ NiTi archwires, for the same charging and ageing conditions, present a superelastic behaviour. We notice only an increase in the critical stress for martensite start stress by 50 MPa and 70 MPa, respectively, compared to the cycled specimen for 13 cycles without immersion. It is important to mention that the tested specimen with different strain rates after 13 cycles with 10^−4^s^−1^ and 10^−3^s^−1^ and immersion does not show any embrittlement during the tensile tests with different strain rates. The TDA after 13 cycles and immersion with the current density of 10 A/m^2^ at different imposed strain rates is represented in Figure 9b. Despite the important amount of absorbed hydrogen of 411 and 543 mass ppm, it is clear that the mechanical behaviour of the NiTi alloy is not remarkably affected during the tensile tests with10^−4^s^−1^ and 10^−3^s^−1^. After 18 cycles of loading and unloading with 10^−3^s^−1^ of the imposed strain rate and hydrogen charging and aging for one day, the tensile test with 10^−5^s^−1^ and 10^−4^s^−1^ shows an embrittlement in the Luder-like plateau of the austenite martensite transformation, as depicted in Figure 10a. Nevertheless, the tensile tested NiTi archwire with a higher imposed strain rate of 10^−3^s^−1^ (corresponding to the shorter time) conserves its superelasticity. This specimen presents a higher martensite start stress and in addition to the lower total strain compared to the non-charged ones. Figure 10a illustrates the TDA after 18 cycles at 10^−3^s^−1^ and hydrogen charging in comparison with loaded specimens at 10^−5^s^−1^ after 13 cycles, where the first embrittlement has happened. It can be seen that the amount of absorbed hydrogen after 18 cycles at 10^−3^s^−1^ (584 mass ppm) is almost twice the one cycled with 10^−5^s^−1^ (291 mass ppm), and no fracture has been detected yet (Figure 10b). Table 1 summarizes the results obtained forall performed experimental conditions. It is important to mention that the experimental results obtained after 20 cycles are similar to the ones given with 18 cycles. Accordingly, the hydrogen quantity may not be the only principal factor while considering in particular the studied superelastic NiTi alloy’s hydrogen embrittlement mechanism.

The strain rate response of the superelastic NiTi alloy can be explained based on a thermo-mechanical phenomenon [44,45]. For a low applied strain rate (10^−5^s^−1^ and 10^−4^s^−1^), once the first variants are generated, the austenite-martensite interfaces move slowly enough in order to enable the latent transformation to be transferred quickly outside. Temperature change in the specimen is eliminated and the transformation is considered under an isothermal process. Consequently, the flat Luder-like plateau of the austenite martensite transformation results from the growth of a few number of martensite bands, not the nucleation of new martensite variants. In a high strain rate of 10^−3^s^−1^, the latent heat cannot be timely convected to the environment since the martensite variants are forced to propagate at a somewhat higher velocity. For the domain-front growth, the domain-fronts’ localized self-heating raises the stress [46], which becomes more important than the untransformed parent zone’s domain-nucleation stress. Therefore, during the phase transformation, the variation in the average sample temperature is significant. The slope of the stress–strain curve goes up and new domains nucleate to fulfill the applied strain, with the continuously changing temperature.

The maximum number of martensite variants $n_{max}$ (or the minimum domain spacing) increases as a function of the strain rate. In fact, the maximal number of martensite domains $n_{max}$ and as well as the strain rate $\dot{\varepsilon }$ canbe fitted into a power-law relationship [47,48]:

(1) $$n_{max}=c\;{\left(\dot{\varepsilon }\right)}^{x},$$

where the fitting coefficient *x* is an exponent factor that is around 0.5, and c is a fitting coefficient.

Hao Yu et al. [49] showed that during a high strain rate, the precipitation of Ni_4_Ti_3_ is found to increase the NiTi yield strength, which is caused by the impediment of Ni_4_Ti_3_ precipitation to dislocation motion and leading to an increase in the martensite phase nucleation at the interface between the precipitation and the B2 matrix. This indicates that during the tensile tests with the same applied strain rate, the number of martensite variants, obtained in the plateau of austenite martensite transformation, should increase with the imposed strain rate during cycling.

The sensitivity to the strain rate after cycling at different strain rates and after hydrogen charging for 3 h and ageing at room temperature appears to be as follows. After 13 cycles with a 10^−5^s^−1^ strain rate of, the 291 mass ppm of absorbed hydrogen seems to represent the critical amount to cause fracture during the austenite martensite plateau. Indeed, during tensile loading with 10^−5^s^−1^, a few martensite variants tend to grow during the austenite-martensite transformation plateau, since the latent transformation heat is transferred rapidly to ambient media. However, this growth is hindered by the trapped hydrogen into the retained martensite bands and the local dislocation stress field near the austenite-martensite interfaces and this actually happens during the repeated martensitic transformation as well as during its reverse. Thus, the growth of martensite bands cannot overcome the obstacle induced by hydrogen. It is important to point out that during tensile loading with 10^−5^s^−1^, the number of martensite variants growing in the plateau for austenite martensite transformation obtained after 13 cycles with an imposed strain rate of 10^−4^s^−1^ and 10^−3^s^−1^ should be more important than after cycling with 10^−5^s^−1^, since we have more local stress near the austenite-martensite interfaces [50]. However, an embrittlement occurs. Thus, it is assumed that the amount of absorbed hydrogen of 543 mass ppm is sufficient to act as an obstacle for the short travelling distance of martensite bands to cause fracture. This points out that there is a competition between the number of martensite variants and the amount of absorbed hydrogen to cause fracture.

With an imposed 10^−3^s^−1^ strain rate, after 18 cycles, a premature fracture is detected for a loaded tensile NiTi alloy with both 10^−5^s^−1^ and 10^−4^s^−1^ strain rates. The corresponding amount of absorbed hydrogen, after 3 h of immersion, is about 584 mass ppm, which is considered as the critical value causing earlier fracture during the monotonic tensile test with a 10^−4^s^−1^ strain rate. The delay of embrittlement with 10^−4^s^−1^ can be related to a competition between the number of martensite bands in the austenite martensite plateau and the quantity of absorbed hydrogen. In fact, after13 cycles, the number of nucleated variants is higher than 10^−5^s^−1^. Consequently, the amount of absorbed hydrogen is not enough to act as an obstacle for the variant growth caused by 10^−4^s^−1^, since the travelled distance is less important than the one obtained with 10^−5^s^−1^. However, after 18 cycles, despite the increase in nucleated bands, and due to (i) the multiplication of local stress near the austenite-martensite interfaces after cycling with 10^−3^s^−1^ and (ii) the higher applied strain rate during tensile test, the quantity of absorbed hydrogen seems to be sufficient to act as an obstacle for varied growth in the quasi-flat plateau. This explanation seems to be in coherence with the obtained superelastic behaviour after 18 cycles and the tensile test with a higher imposed strain rate. In this case, the time for transferring the latent heat is highly restricted, causing the nucleation of a large number of martensite bands with small domain spacing between them. As a result, the travelled distance of these martensite bands will be very limited and the amount of absorbed hydrogen will have no effect.

## 4. Conclusions

We have investigated in this study the effects of the strain rate response after hydrogen charging for 3 h and ageing for one day on pre-cycled superelastic NiTi archwires. Cycling from 1 to 50 loading and unloading at 10^−5^s^−1^, 10^−4^s^−1^, and 10^−3^s^−1^ has been carried out to generate a gradual rise in the residual strain at zero stress. Moreover, cycling, which is characterized by an increase in the density of dislocation and retained martensite after unloading, represents a preferential site for hydrogen diffusion during immersion. After hydrogen charging with 191 mass ppm of absorbed hydrogen, an embrittlement occurs for the low applied tensile strain rate. In addition, this fracture happens only with a higher quantity of absorbed hydrogen (584 mass ppm) with a 10^−4^s^−1^ imposed strain rate, and no embrittlement will be detected for the higher strain rate of 10^−3^s^−1^. We attribute this behaviour to the interaction between the travelled distance during the increase in martensite bands and the amount of absorbed hydrogen during the step of austenite-martensite transformation.

## Figures and Tables

**Table 1:** Summary of conducted tensile tests at different strain rates after immersion and cycling with 10^−5^s^−1^, 10^−4^s^−1^ and 10^−3^s^−1^.

	N^bre^ of Cycles	13 Cycles			18 Cycles		
Cyclic Strain Rate		Absorbed Hydrogen (Mass ppm)	Tensile Strain Rate	Fracture	Absorbed Hydrogen (Mass ppm)	Tensile Strain Rate	Fracture
10^−5^s^−1^		291	10^−5^s^−1^	F	313	10^−5^s^−1^	F
			10^−4^s^−1^			10^−4^s^−1^	
			10^−3^s^−1^			10^−3^s^−1^	
10^−4^s^−1^		411	10^−5^s^−1^	F	436	10^−5^s^−1^	F
			10^−4^s^−1^			10^−4^s^−1^	
			10^−3^s^−1^			10^−3^s^−1^	
10^−3^s^−1^		543	10^−5^s^−1^	F	584	10^−5^s^−1^	F
			10^−4^s^−1^			10^−4^s^−1^	F
			10^−3^s^−1^			10^−3^s^−1^	

F: Fracture.

## Data Availability

The data presented in this study are available on request from the corresponding author.
